# Ischemic Muscle Necrosis of Lower Extremities in Peripheral Arterial Disease: The Impact of 99mTc-MDP Scintigraphy on Patient Management

**DOI:** 10.3390/medicina55120763

**Published:** 2019-11-28

**Authors:** Donatas Jocius, Donatas Vajauskas, Arminas Skrebunas, Marijus Gutauskas, Algirdas Edvardas Tamosiunas

**Affiliations:** 1 Vilnius university, Faculty of medicine, Vilnius 03101, Lithuania; arminas.skrebunas@santa.lt (A.S.); marijus.gutauskas@santa.lt (M.G.); algirdas.tamosiunas@santa.lt (A.E.T.); 2 Vilnius University Hospital Santaros Klinikos, Radiology and Nuclear Medicine Center, Vilnius 08410, Lithuania; 3 Lithuanian University of Health Science Kauno Klinikos, Department of Radiology, Medical Academy, Kaunas 50161, Lithuania; vajauskas@yahoo.com; 4 Vilnius University Hospital Santaros Klinikos, Vascular Reconstruction and Endovascular Surgery Center, Vilnius 08410, Lithuania

**Keywords:** peripheral arterial disease, ischemic lower extremity, scintigraphy, 99mTc-MDP, perfusion

## Abstract

*Background and objectives:* The objective of this study was to assess the value of a whole-body bone scintigraphy using 99m technetium labelled-methyl diphosphonate (99mTc-MDP) for the diagnosis and the assessment of grades of muscle damage after prolonged acute or chronic obstruction of the main arteries in lower extremities. *Material and Methods:* Fifty consecutive patients were selected for the study. The patients’ condition had not improved after primary peripheral arterial reconstruction operation or limb amputation and after conservative treatment. The clinical suspicion was of arterial obstruction and muscle necrosis. All the patients underwent whole-body scintigraphy with 99mTc-MDP. Muscle necrosis was identified as an increased soft tissue uptake of 99mTc-MDP. *Results:* Forty-five patients had gross muscle necrosis detected on whole-body scintigraphy with 99mTc-MDP and were histologically confirmed after repeated surgery (necrectomy or amputation) or by muscle biopsy, if only fasciotomy was performed. The location and extent of muscle injury were assessed preoperatively and the findings were confirmed in all 45 patients. Twelve patients with clinically suspected minor muscle damage, which was confirmed as relatively minor muscle necrosis on 99mTc-MDP scintigraphy, were treated conservatively. The clinical outcome of all 50 patients was favorable. The 99mTc-MDP scintigraphy, in detection of muscular necrosis, demonstrated sensitivity, specificity, and accuracy of 97.3% (95% confidence interval (CI) 85.4 to 99.3%), 30.77% (95% confidence interval (CI) 9.09 to 61.43%), and 80% (95% confidence interval (CI) 66.28 to 89.97%), respectively. *Conclusion:* The 99mTc-MDP scintigraphy is a valuable tool in the detection of muscular necrosis. It is able to define location, extent, and grade of involvement. Therefore, it has a clinical impact in patient management, allowing clinicians to select adequate treatment policy and specify the scope of necrectomy.

## 1. Introduction 

Acute or chronic arterial insufficiency may cause prolonged periods of ischemia in limb tissues. In these situations, despite continued improvements in surgical vascular reconstruction techniques, tissue necrosis may develop. Currently, the extent of muscle damage and viability are assessed by clinical examination, which is frequently inaccurate. Accurate and objective assessment of necrotic tissue for early detection is valuable in the management of such patients and can have a significant impact on improving morbidity and mortality [1]. Nevertheless, patients with arterial obstructive lower extremity disease are also at high intraoperative risks due to coronary or cerebral artery obstruction and, therefore, any surgery performed should be confined to a single occasion in order to prevent further intervention [2].

The diagnosis of peripheral vascular disease is usually established on the basis of clinical findings and contrast angiography data [3]. Magnetic resonance imaging may also be used to assess the necrosis of muscles. However, these non-invasive methods are limited because they cannot assess tissue perfusion and the extent of necrosis of ischemic peripheral muscles at a cellular level.

Although 99m technetium labelled-methyl diphosphonate (99mTc-MDP) is customarily used to evaluate skeletal pathologic conditions, its excellent clearance from normal soft tissues allows the detection of abnormal 99mTc-MDP accumulation [4]. Mechanisms leading to increased extra-osseous 99mTc-MDP uptake include extracellular fluid expansion, enhanced regional vascularity and permeability, and elevated intracellular calcium concentration. The composition of the calcium deposition and the presence of other metallic ions are important [4]. Ischemic damage to cellular membranes results in the rapid intracellular influx of calcium, which precipitates as a salt within the mitochondria. Denatured proteins further act as substrates for calcium deposition, with an affinity for 99mTc-MDP. Therefore, the increased 99mTc-MDP uptake in the muscles reveals the extent of ischemic tissues damage.

As muscle damage is the critical determinant for clinical outcome in patients with advanced peripheral arterial disease, we tried to apply scintigraphy as a non-invasive method for the preoperative detection of muscle necrosis.

## 2. Materials and Methods 

### 2.1. Patients 

Fifty patients’ clinical data were retrospectively reviewed. All the patients were treated at Vilnius University Hospital Santaros Klinikos between December 2012 and February 2017. All patients who were referred to the nuclear medicine department for 99mTc-MDP whole-body scintigraphy were included in this study without any other inclusion criteria. Vilnius Regional Bioethics Committee approved this retrospective study (Approval No. 158200-18/10-1066-566).

The limb muscle necrosis was suspected due to elevated serum myoglobin levels and clinical symptoms of rhabdomyolysis. Forty patients (80.0%) had previous primary peripheral arterial reconstruction operation due to arterial obstruction or limb amputation, if arterial reconstruction was impossible. In case their conditions had not improved and the levels of potassium, urea, and C-reactive protein (CRP) were still increasing, muscle necrosis of the lower extremity was suspected. Additionally, 10 patients were enrolled with the following conditions: 4 (8.0%) patients with positional compression syndrome, 4 (8.0%) patients after traumatic injury of the major vessels, and 2 (4.0%) patients after vascular surgery (kidney transplant and abdominal aorta aneurism surgery). All 10 patients had clinical suspicion of muscular necrosis.

### 2.2. Scintigraphy 

All 50 patients underwent 99mTc-MDP whole-body scintigraphy and single-photon emission computed tomography (SPECT) using a dual head GE Hawkeye gamma camera, equipped with low-energy, general-purpose collimators. Injected activity of 99mTc-MDP to the patients was 490.0 (± 85.9) MBq. 

For the whole-body scan, patients were positioned supine on the gamma camera table. Whole-body scans were acquired as early as 10 min post-injection and delayed 2 hours post-injection at a 10 cm per minute continuous scanning rate, followed by SPECT in the areas of observed rhabdomyolysis for delineation and to determine the extent of muscle necrosis. SPECT acquisition parameters were: 360° circular orbit, 60 steps, 128 x 128 matrix, and 30 s per stop.

Hybrid SPECT/CT (single-photon emission computed tomography fused with low-dose computer tomography) imaging was performed in 22 patients, in the region of observed rhabdomyolysis, for anatomical delineation of 99mTc-MDP uptake in affected muscle groups.

### 2.3. Image Analysis 

**Figure 1:**
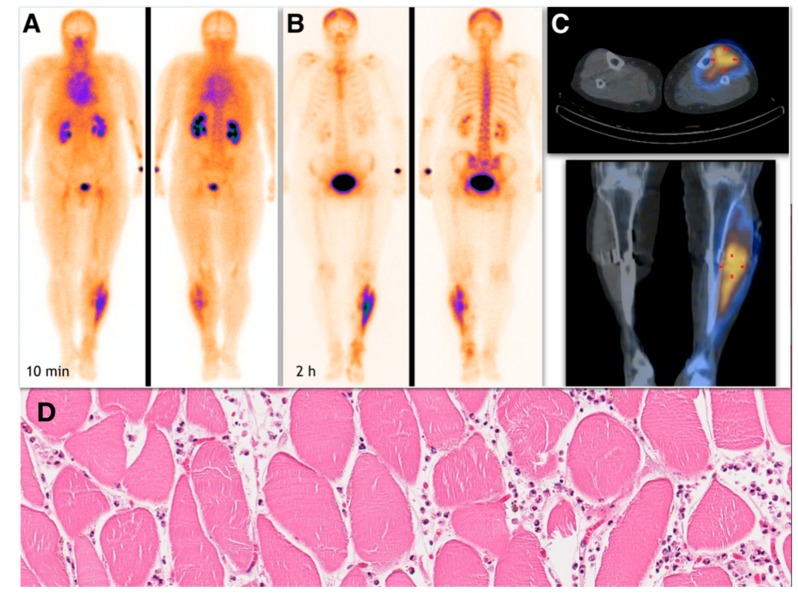
Whole-body 99m technetium labelled-methyl diphosphonate (99mTc-MDP) scintigraphy. Early scan performed at 10 min post-injection (**A**) and delayed scan performed at 2 hours post-injection (**B**). SPECT/CT scan (**C**). Intensive (grade 2) uptake with clear delineation of necrosis-affected muscle is seen in the left shin muscle groups (tibialis anterior and posterior muscles and extensor digitorum longus muscle). Below, there is a corresponding HE-stained pathological image representing muscle necrosis with surrounding white blood cells infiltration (**D**). With permission of J. Drachneris, MD (National Center of Pathology, Vilnius, Lithuania).

Muscle necrosis was identified if increased 99mTc-MDP uptake was seen on scintigraphic images in soft tissues corresponding to the muscle group (Figure 1). 

**Figure 2:**
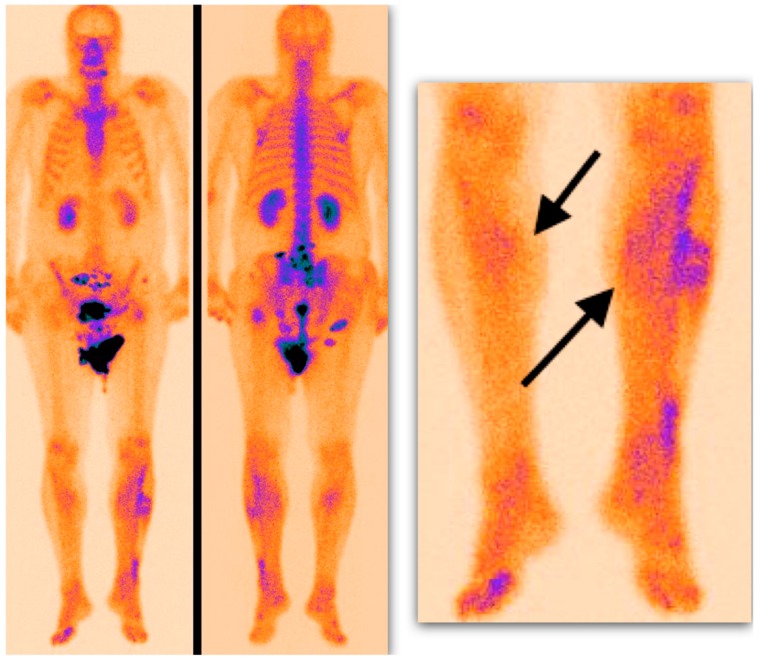
Delayed whole-body 99mTc-MDP scintigraphy. Mild, diffuse soft tissue uptake is demonstrated in the shins (arrows) (grade I uptake).

**Figure 3:**
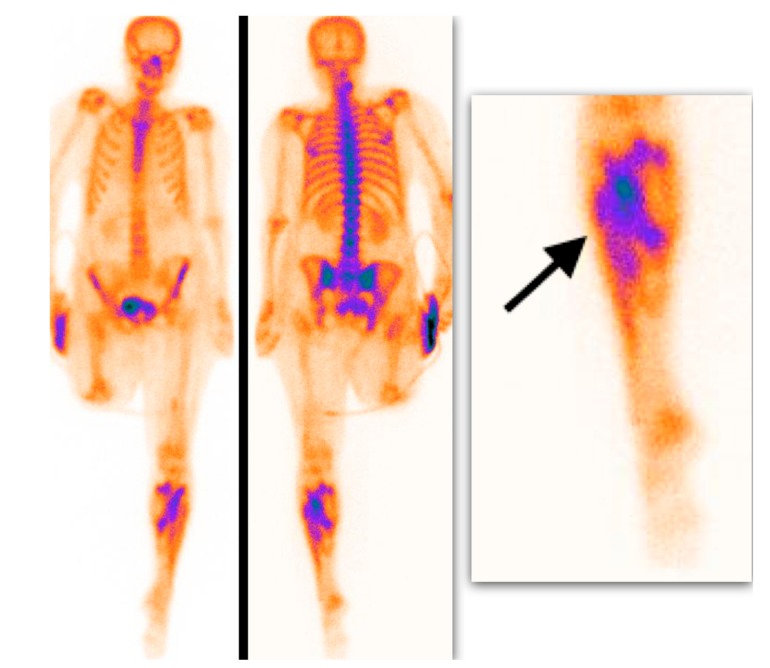
Delayed whole-body 99mTc-MDP scintigraphy. Intensive muscle uptake is shown in the left shin (arrow) (grade II uptake).

**Figure 4:**
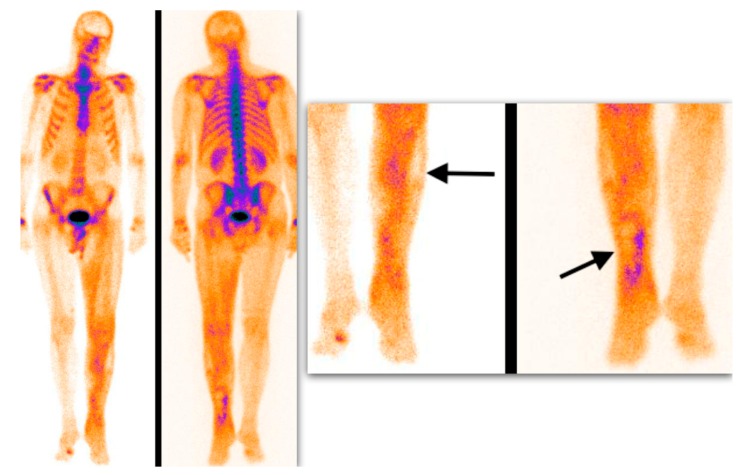
Delayed whole-body 99mTc-MDP scintigraphy. Uptake defect surrounded by intensive muscle uptake (arrows) is presented (avascular area, grade III uptake).

Semiquantitative analysis of 99mTc-MDP scintigraphy was performed accordingly. Mild soft tissue uptake equal to the adjacent long-bone uptake (grade I), with or without possibility to delineate muscle groups on SPECT or SPECT/CT images, was considered a soft tissue edema with silent muscle necrosis (Figure 2). Intensive muscle uptake 2–3 times higher than the adjacent long-bone uptake (grade II), with clear muscle group delineation on SPECT or SPECT/CT images, was considered rhabdomyolysis (Figure 3). Uptake defect (photopenic uptake area) within the intensive muscle uptake was seen in the avascular area of the muscle surrounded by muscle necrosis (grade III), with clear muscle group delineation on SPECT or SPECT/CT images (Figure 4).

### 2.4. Statistical Analysis 

Statistical analysis was performed in R statistical package (version 2.4-4, Auckland, New Zeland). Data was checked for normality using the Shapiro–Wilk test and expressed as mean ± one standard deviation or median and interquartile range for normal and non-normal data distribution, respectively. The contingency coefficient was calculated to determine the relationship between whole-body 99mTc-MDP scintigraphy findings and treatment decision.

The null hypothesis was rejected when the *p*-value was less than 0.05.

## 3. Results 

Clinical data and whole-body 99mTc-MDP scintigraphy data were analyzed retrospectively in 38 males (76.0%) and 12 females (24.0%) with a mean age of 75 ± 6.5 (range: 64–85) years. Thirty-one patients (62.0%) were hospitalized for acute ischemia of the lower extremity and 19 (38.0%) for chronic ischemia.

The diagnosis of muscle necrosis at first presentation was made according to general condition and increased potassium, urea, and CRP levels before the first operation. Primary interventions after the initial diagnosis of muscle necrosis included amputation in 18 patients (36%), shunting procedure in 10 patients (20%), thrombectomy in 17 patients (34%), fasciotomy in 4 patients (8%), and only 1 patient (2%) was left for follow-up (Table 1).

**Figure 5:**
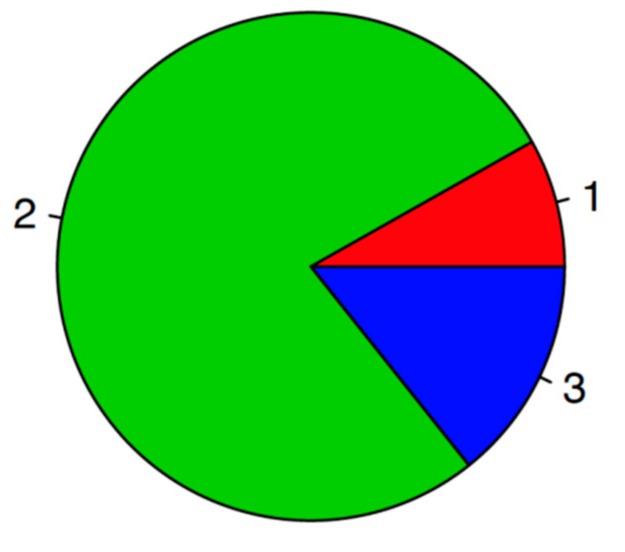
Uptake categories of the study population: 1, grade I uptake; 2, grade II uptake; 3, grade III uptake.

99mTc-MDP scintigraphy was performed in all 50 patients (including non-operated patients) after primary treatment to assess the ischemic muscle damage in the lower extremity. The intramuscular uptake of 99mTc-MDP on whole-body scans was categorized into three categories according to the relative uptake. The findings of five patients corresponded to grade 1 (10.0%), 38 patients to grade 2 uptake (76.0%), and 7 patients to grade 3 (14.0%) (Figure 5).

The median interval between the first operation and scintigraphy was 5 ± 3.1 (range: 1–10) days. Gross muscle necrosis (grade II or III uptake) was detected on scintigraphic scans in 45 patients (90.0%). The scintigraphic findings and the diagnosis of rhabdomyolysis were confirmed either by muscle biopsy, if only fasciotomy was performed (2 patients or 4.0%), or by histological examination of surgical specimen: amputation in 15 patients (30.0%); necrectomy in 14 patients (28.0%); and exarticulation of the lower extremity in 7 patients (14.0%).

**Figure 6:**
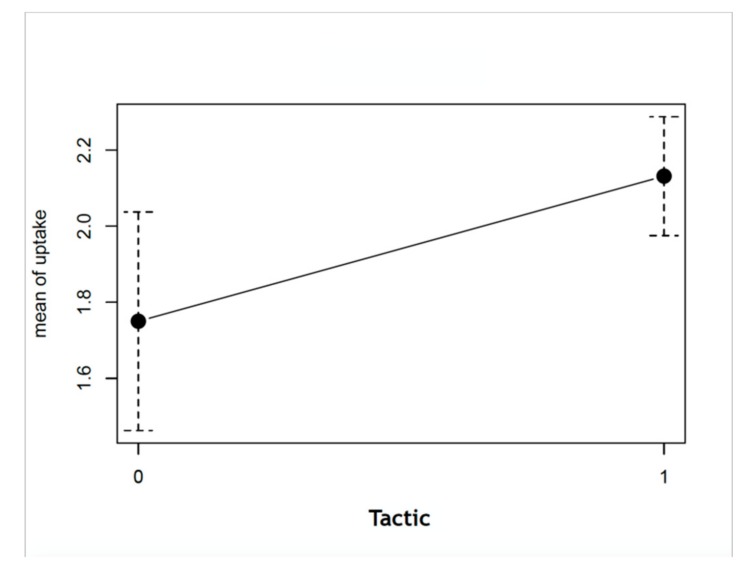
Difference in mean uptake grades between conservative and aggressive treatment tactics (0, conservative treatment; 1, aggressive treatment; *p*-value - 0.02081).

Two groups of patients were defined, according to second-line treatment tactical decisions, into conservative (n - 12) (best conservative therapy: fasciotomy and necrectomy) and aggressive (n - 38) (shunting or amputation) groups. None of the patients in the conservative group had grade III 99mTc-MDP uptake; the majority of the patients in both groups had uptake grade II (Table 2). The mean difference in grades between the conservative and aggressive treatment groups was found to be statistically significant (*p*-value - 0.02081; Figure 6).

Due to clinically suspected minor muscle damage, good clinical status, and relatively minor (prevailing uptake grade I–II) muscle necrosis shown by the 99mTc-MDP scintigraphy, 12 patients (24.0%) were applied the best conservative therapy including fluid infusions, anticoagulation, antibiotic therapy, analgesics, and fasciotomy, if needed.

To detect the relationship between the uptake category and treatment tactics, we compared treatment tactics (conservative versus aggressive) in-between uptake categories and found a positive, moderate relationship (contingency coefficient was 0.322 and *p*-value of Fisher’s exact test was 0.04597) suggesting that treatment decision could depend on intramuscular MDP uptake.

We also divided the uptake categories into two groups (grade 1 and grade 2, 3 groups) according to the expected treatment decision. Although we did not find a significant difference (*p*-value of the Fisher’s exact test was 0.08225), this is a borderline result and could be somewhat different if the sample size of the study was bigger.

The clinical outcome was favorable for all 50 patients. Potassium, urea, and CRP levels decreased after the second operation (Table 3). However, there was no statistically significant difference between blood test data before and after re-operation. Sensitivity, specificity, positive predicting values, negative predicting value, and accuracy are presented in Table 4.

All the patients were discharged in satisfactory condition.

## 4. Discussion 

Acute or chronic arterial insufficiency may cause prolonged tissue ischemia periods in the lower extremities resulting in tissue necrosis. In such situations, surgical treatment is often required to improve blood flow to the extremity or to remove non-viable tissues. Besides knowledge of the vascular anatomy, functional and biochemical data are important in choosing treatment options for six peripheral arterial diseases [5]. Collateral circulation and microcirculation are the major determinants of tissue viability in the ischemic limb [6,7,8]. Therefore, it may be difficult to assess the exact and significant hemodynamic changes using traditional contrast angiography or Doppler ultrasound imaging of the limb if arterial stenoses or occlusions are present. These methods show blood flow only in large and intermediate vessels and do not asses microvascular changes. It is also necessary to determine limb perfusion for soft tissues. Nuclear medicine imaging with 99mTc-MDP may asses collateral blood flow, microcirculation, and could represent the perfusion of the muscles. In addition to that, 99mTc-MDP scintigraphy could represent cellular changes when tissue ischemia is present.

Skin blood flow and skin perfusion pressure using various nuclear medicine imaging techniques have been reported to provide valuable data for tissue ischemia [9,10,11,12]. However, these methods do not reflect perfusion to deeper tissues and do not indicate the extent or distribution of muscle damage [1]. They cannot be efficiently applied to evaluate ischemic muscle necrosis.

Based on myocardial perfusion scintigraphy, some investigators have found nuclear medicine imaging to be useful for the assessment of peripheral muscles perfusion [13,14]. Several studies have demonstrated that three phase bone scintigraphy with 99mTc-MDP can be applied for the evaluation of tissue ischemia degree [15,16] after prolonged acute or chronic obstruction in the main arteries of the lower extremity. However, clinical use of these methods is limited.

As noted by Bhatnagar et al., 99mTc pertechnetate accumulates in the extracellular fluid and provides only indirect evidence on tissue viability [17]. The results of blood flow measurements with 99mTc pertechnetate are not always reproducible and reliable, and determination of viability at the cellular level is doubtful with this method [18,19]. 99mTc-sestamibi has been found to be an appropriate radionuclide agent for the imaging of perfusion of the lower extremities [20,21,22,23,24,25,26,27,28,29,30]. Its cellular uptake and trapping are related not only to regional blood flow but also to mitochondrial metabolic conditions and viability [31]. The tracer is distributed across both plasma and mitochondrial membranes in response to progressively larger negative transmembrane potentials, and trapped within the mitochondrial layer [32]. In a study by Timmons et al., 99mTc pyrophosphate scintigraphy allowed accurate prediction of the location of necrotic muscle [33].

In our study, we assumed that there are advantages of 99mTc-MDP scintigraphy, which could be utilized in lower limb scintigraphy, because 99mTc-MDP has rapid blood clearance, excellent in vivo chemical stability, and a high bone-to-soft tissue ratio [34] allowing the detection of abnormal extraosseous 99mTc-MDP uptake if muscle damage is present. 99mTc-MDP uptake in normal tissues is proportional to their calcium content [35]. The increased calcium level in damaged skeletal muscle strongly correlates with 99mTc-MDP uptake concentration [36,37]. Ischemic damage to cellular membranes results in the rapid intracellular influx of calcium, which has a high affinity to 99mTc-MDP [4]. 99mTc-MDP locates in irreversibly damaged or dying muscle cells if some residual blood flow is present.

In the present study, 99mTc-MDP scintigraphy images and recommended different uptake grades revealed increased soft tissue uptake in the lower limbs in all 50 patients with advanced lower extremities ischemia. Nuclear medicine imaging data confirmed the clinically suspected ischemic muscle damage.

The findings of our study show that grade II–III uptake derived from scintigraphic imaging represents gross muscle necrosis, clinically directing towards more aggressive surgical treatment such as necrectomy, amputation, or exarticulation. Grade 1 uptake, representing only minor muscle damage, indicates that a more conservative treatment approach could be employed including infusion therapy, anticoagulant therapy, antibiotic therapy, analgesics, and fasciotomy, if needed.

Intraoperatively removed tissues or tissue biopsy specimen were sent for histological examination and muscle necrosis was confirmed in all cases. Patients’ general health and blood test results improved after additional treatment.

Another interesting finding, which needs to be further clarified, is that the majority of patients (76.0%, n - 38) required second-line surgical treatment after primary surgery, the treatment decision for which was made using traditional methods. This finding may suggest that primary treatment is not sufficient enough to achieve the best result and the cause of this could be non-radical surgical removal of only a part of necrotic muscles, by which the extent is decided based only on clinical signs, or even by the selection of non-optimal surgical tactics. The exact delineation of affected muscles before primary operation may produce better results comparing to only clinically-based decision and this could be achieved using 99mTc-MDP scintigraphy.

Although the evaluation of peripheral vascular disease with 99mTc-MDP scintigraphy is very appealing because of the relatively small number of scans performed, its value cannot be considered to have been completely proven by this study. However, our experience shows that 99mTc-MDP imaging is a promising adjunctive diagnostic technique for decision making in treatment of advanced peripheral vascular disease.

Further, prospective studies should be planned to evaluate this technique in larger patients’ populations and in more homogenous groups of patients with suspected muscle necrosis.

The limitations of this study include its retrospective manner, non-selected patient population, and a relatively small sample size. In addition to that, 99mTc-MDP scintigraphy was not directly compared with traditional and other potential imaging methods.

## 5. Conclusion 

The 99mTc-MDP scintigraphy is a non-invasive, reliable, and safe diagnostic method for assessing ischemic muscle damage after prolonged acute or chronic obstruction of the main arteries of the lower extremity. This is the first attempt to categorize muscular necrosis in patients depending on 99mTc-MDP uptake, consequently leading to selection of the most appropriate treatment, the best being conservative therapy in patients with grade I uptake and major surgeries (necrectomy, amputation, or exarticulation) in patients with grade II and III uptake.

## Figures and Tables

**Table 1:** Primary treatment decision.

Primary Treatment	Amputation	Fasciotomy	Shunting	Thrombectomy	None
	18 (36%)	4 (8%)	10 (20%)	17 (34%)	1 (2%)

**Table 2:** Uptake types in conservative and aggressive patient groups.

	Grade I	Grade II	Grade III
**Conservative**	3	9	0
**Aggressive**	2	29	7

**Table 3:** Potassium, urea, and C-reactive protein (CRP) levels before and after re-operation.

	Before Re-Operation	After Re-Operation	*p*-Value
K, mmol/L	3.91 ± 0.43	3.90 ± 0.81	0.973
Urea, mmol/L	12.0 ± 9.4	4.7 ± 1.3	0.207
CRP, mg/L	142.9 ± 99.0	84.8 ± 77.8	0.142

**Table 4:** 99mTc-MDP scintigraphy sensitivity, specificity, positive predicting values, negative predicting value, and accuracy.

Statistical Measure	Percentage	95% CI
Sensitivity	97%	85.84 to 99.93%
Specificity	31%	9.09 to 61.43%
Positive Predictive Value	80%	73.50 to 85.23%
Negative Predictive Value	80%	32.92 to 97.02%
Accuracy	80%	66.28 to 89.97%
